# Chronic *Mycobacterium avium* infection differentially affects the cytokine expression profile of three mouse strains, but has no effect on behavior

**DOI:** 10.1038/s41598-023-33121-2

**Published:** 2023-04-17

**Authors:** Susana Roque, Daniela de Sá-Calçada, Bruno Cerqueira-Rodrigues, Susana Monteiro, Susana G. Guerreiro, Joana A. Palha, Margarida Correia-Neves

**Affiliations:** 1 Life and Health Sciences Research Institute (ICVS), School of Medicine, University of Minho, Braga, Portugal; 2 ICVS/3B’s-PT Government Associate Laboratory, Braga/Guimarães, Portugal; 3 Institute for Research and Innovation in Health (i3S), Porto, Portugal; 4 Institute of Molecular Pathology and Immunology of the University of Porto-IPATIMUP, Porto, Portugal; 5 Biochemistry Unit, Department of Biomedicine, Faculty of Medicine, University of Porto, Porto, Portugal; 6 Division of Infectious Diseases, Department of Medicine Solna, Karolinska Institutet, Stockholm, Sweden

**Keywords:** Neuroimmunology, Infection

## Abstract

One of the most remarkable findings in the immunology and neuroscience fields was the discovery of the bidirectional interaction between the immune and the central nervous systems. This interplay is tightly regulated to maintain homeostasis in physiological conditions. Disruption in this interplay has been suggested to be associated with several neuropsychiatric disorders. Most studies addressing the impact of an immune system disruption on behavioral alterations focus on acute pro-inflammatory responses. However, chronic infections are highly prevalent and associated with an altered cytokine *milieu* that persists over time. Studies addressing the potential effect of mycobacterial infections on mood behavior originated discordant results and this relationship needs to be further addressed. To increase our understanding on the effect of chronic infections on the central nervous system, we evaluated the role of *Mycobacterium avium* infection. A model of peripheral chronic infection with *M. avium* in female from three mouse strains (Balb/c, C57BL/6, and CD-1) was used. The effect of the infection was evaluated in the cytokine expression profile (spleen and hippocampus), hippocampal cell proliferation, neuronal plasticity, serum corticosterone production and mood behavior. The results show that *M. avium* peripheral chronic infection induces alterations not just in the peripheral immune system but also in the central nervous system, namely in the hippocampus. Interestingly, the cytokine expression profile alterations vary between mouse strains, and are not accompanied by hippocampal cell proliferation or neuronal plasticity changes. Accordingly, no differences were observed in locomotor, anxious and depressive-like behaviors, in any of the mouse strains used. We conclude that the *M. avium 2447* infection-induced alterations in the cytokine expression profile, both in the periphery and the hippocampus, are insufficient to alter hippocampal plasticity and behavior.

## Introduction

An increasing body of evidence robustly points towards a bidirectional interaction between the immune and the central nervous systems. Behavioral alterations are often associated with an imbalance in the immune system, namely in the cytokine profile^1–4^. Since infections (acute and chronic) are very frequent and induce alterations in the immune system, including increasing the production of pro-inflammatory cytokines, it is reasonable to infer their contributive and potential role in the pathophysiology of mood disorders. In fact, lipopolysaccharide (LPS, a cell wall component of Gram-negative bacteria) administration or *Salmonella typhi* vaccines in mice induce a pattern of behavioral changes that share similarities with those observed in mood disorders^5–9^. These behavioral alterations, collectively termed “sickness behavior,” result from the interaction between the immune, endocrine, and central nervous systems as a normal acute physiological response to a danger signal, which is particularly important for host survival and infection clearance. Of note, the acute behavioral alterations are rapidly restored with the normalization of the immune system balance, which occurs a few days after the triggering stimuli. Interestingly, few reports addressed chronic infections’ effect on mood disorders. Chronic infections are of particular interest since they are widely prevalent and are accompanied by a sustained altered cytokine profile.

Mycobacterial infections are among the major health threats worldwide^10^. *Mycobacterium avium* induces a chronic infection, altering the production of many pro-inflammatory cytokines, thus representing an attractive model to address the interplay between a chronically altered cytokine profile and behavior. *M. avium* is an opportunistic microorganism, frequently present in the environment, that causes serious disseminated infection in immunosuppressed patients^11,12^. The mouse model of *M. avium* infection is very well characterized^13^. The infection can last several months with no obvious clinical signs of disease^14^. Moreover, *M. avium* infection has been extensively used to address specific questions related to this infection and, as a mouse model, to study general features of mycobacterial infections^13^. The infection of mice with *M. avium 2447* is associated with an increase in the bacterial load until 4 weeks post infection (wpi) in organs such as spleen and liver^15–18^. At 4 wpi the bacterial load reaches a plateau that is preserved for several months (previous studies described until 1 year) in organs such as spleen, liver and lung^15^. Even though the host immune system can halt the bacterial growth in those organs, the *M. avium* 2447 bacterial load is not reduced or eliminated. Upon *M. avium* 2447 infection, an immune response is continuously present, characterized mostly by a Th1 type of response, with increased levels of pro-inflammatory cytokines such as IFN-γ and TNF^18^, reaching a peak of the immune response at 4 wpi^15–18^.

The effect of mycobacterial infections on behavior has been previously studied by others, however, results from these studies are discordant. Several reports have showed comorbidity between mental disorders (such as depression and anxiety) and tuberculosis^19–21^. On the other hand, clinical trials with *Mycobacterium vaccae* treatment have been undertaken in patients with allergic disorders, psoriatic arthritis and some types of cancer, and it was reported to improve the quality of life scores in these patients^22–24^. In animal models, studies performed with *Mycobacterium bovis* bacillus Calmette-Guérin (BCG) infection suggested that mycobacterial infection induces depressive-like behavior in CD1^25–27^, C57BL/6^26,28,29^ and Balb/c mice^30,31^. In contrast, stimulation of the immune system with antigens from *M. vaccae*, using Balb/c mice, led the authors to conclude that mycobacterial antigens “decrease” depressive-like behavior^32^. Moreover, this immune challenge with *M. vaccae* also conferred stress resilience in mice and rats^33–36^ and enhanced fear-extinction in rats^37^. The discordant observations between studies with BCG and *M. vaccae* may originate from the different bacteria used. Thus, to further investigate the effect of the immune system imbalance occurred during mycobacterial chronic infections, we evaluated the impact of an infection with *M. avium 2447* using simultaneously female from three mouse strains: two inbred (C57BL/6 and Balb/c) and one outbred strain (CD1). We investigated the brain cytokine expression profile and plasticity and mouse behavior.

## Materials and methods

### Animals

Specific pathogen-free Crl:CD1(ICR), C57BL/6J and BALB/cByJ 8 weeks old female mice were purchased from Charles River Laboratories (Barcelona, Spain). All mice were housed in sterile housing conditions, in groups of 5 per cage, under standard laboratory conditions (12 h light/12 h dark cycle, at 22 °C, relative humidity of 55%; food and water ad libitum). Cages were environmental enriched with a soft paper as nesting material. All experimental procedures were conducted within the light period of the light/dark cycle. Even though no adverse effects were expected in this experimental procedure, mice were monitored and humanely euthanized whenever a clinical sign of a humane endpoint was observed^38^.

### Ethics declaration

The experiments were conducted in agreement with National guidelines (DL 113/2013 and Portaria 1005/92) and with the European Union Directive 2010/63/EU on animal care and experimentation. The study and people directly involved in animal experiments were certified by the Ethical Committee Board of the Portuguese Veterinary Directorate (DGAV). The animal experimental protocol was approved by DGAV (# 015584). The study was performed in compliance with the ARRIVE guidelines^39^.

### Infection and quantification of bacterial load

Mice were randomly assigned to non-infected or infected groups, using a computer based random order generator (Excel v.2301, Microsoft Office 365). In the infected group, mice were infected intravenously (i.v.) through a lateral tail vein, with 10^6^ colony-forming units (CFU) of *M. avium* strain 2447 (smooth transparent variant, provided by Dr. F. Portaels, Institute of Tropical Medicine, Antwerp, Belgium) diluted in saline. Mice in the non-infected group were injected (i.v.) through a lateral tail vein with saline.

The *M. avium* 2447 inoculum was prepared as previously described^40^. Briefly, a colony of bacteria, from previously infected animals, was harvested and grown until mid-log phase in Middlebrook 7H9 liquid medium with 0.04% Tween 80 at 37 °C. Bacteria were centrifugated, suspended in a small volume of saline, and sonicated to break up bacterial clumps. The bacterial suspension was then diluted in saline, frozen in aliquots, and stored at − 80 °C until use. Prior to inoculation, aliquots of bacteria were thawed at 37 °C and diluted in saline to the desired concentration.

At 4 wpi (and 12 wpi—supplementary Fig. S1), mice were submitted to behavioral tests and three days after the end of the behavioral evaluation, animals were weighed and euthanized (mice were anesthetized with a combination of ketamine 75 mg kg^−1^ and medetomidine 1 mg kg^−1^ and lastly euthanized by decapitation, by trained certified personnel). Half of the spleen from all animals and the brain from 8 to 14 mice were collected, homogenized, serially diluted, and plated onto Middlebrook 7H10 agar medium. The number of CFU was counted after 1 week of incubation at 37 °C.

### Behavioral tests

Behavioral tests were performed on 3 consecutive days, between 9 a.m. and 6 p.m., in the following order: open field test (OFT), forced swim test (FST) and tail suspension test (TST). All animals performed all the behavioral tests. The estrous cycle stage (proestrus, estrus, metestrus and diestrus) of each female was determined, immediately after the performance of each behavioral test (in 3 consecutive days), by vaginal smear examination for the presence of leukocytes, cornified epithelial and nucleated epithelial cells and their proportions in the smear^41^. To evaluate the impact of the estrous cycle on the behavioral parameters analyzed, data were grouped into proestrus/estrus and metestrus/diestrus stages since it has been described that the estrogen levels of the grouped stages are very similar^42^. A two-way ANOVA analysis with a post hoc Fisher LSD test revealed that the estrous cycle did not impact the behavioral parameters assessed.

#### Open field test

The OFT was performed to assess locomotor and exploratory activities and anxious-like behavior. Animals were placed in the center of an arena (43.2 × 43.2 cm with transparent acrylic walls and a white floor) and their position and rearings (vertical activity) were monitored and recorded by a three 16-beam infrared system (MedAssociates, VT, USA), during 5 min. The total distance travelled by each animal was used to assess locomotor activity and the number and duration of rearings to determine exploratory behavior. The percentage of time spent in the central area of the OFT arena and the percentage of the distance travelled by each animal in the center of the arena (10.8 cm × 10.8 cm) were used as an indicative measure of anxious-like behavior^43^.

#### Forced swimming test

The FST was used to evaluate the ability of mice to cope with a stressful and inescapable situation (behavioral despair). In this test, each animal was placed in a cylinder (17 cm of diameter and 30 cm of height) filled with water (25 °C) to a depth, so the mouse had no solid support for the rear paws or tail. The activity was recorded for 6 min, and the last 4 min were scored for mobility/immobility. Additionally, the latency to immobility, which corresponds to the time each animal takes from the beginning of the test to stop for the first time, was assessed. Mice displaying decreased latency to immobility and longer immobilization periods were considered to display higher behavioral despair, a sign of depressive-like behavior^44^. The behavior parameters assessed were scored by, at least, two independent researchers, blind to the experimental condition. Since the results are consistent between raters the graphs present data from one rater.

#### Tail suspension test

The TST, as the FST, addresses depressive-like behavior. Mice were suspended by the tail for 6 min. The activity was recorded and, subsequently, the latency, mobility and immobility time were manually scored by at least two independent researchers, blind to the experimental conditions. Since the results are consistent between raters, the data presented in the graphs are from one of the raters. Displaying decreased latency to immobility and longer immobilization periods were considered traits of depressive-like behavior^45^.

### mRNA expression quantification by qPCR

Spleen and hippocampus were macroscopically dissected and stored at – 80 °C for subsequent quantification of messenger RNA (mRNA) expression levels by real-time polymerase chain reaction (qPCR).

To assess the cytokine expression profile in the spleen and hippocampus, the expression levels of genes encoding for several pro- and anti-inflammatory cytokines (*Ifn-γ, Tnf, Il-1β, Il-6, Tgf-β and Il-10*) and for the enzymes *indoleamine 2,3-dioxygenase* (*Ido*) and *inducible nitric oxide synthase* (*iNos*) were measured by qPCR using the primer sequences described in Table 1. Total RNA (1 µg) was reverse transcribed using iScript cDNA synthesis kit (Bio-Rad, Hercules, CA, USA). The geometric mean of the mRNA expression levels of three different genes was used as reference: *hypoxanthine guanine phosphoribosyl transferase* (*Hprt*); *glyceraldehyde 3-phosphate dehydrogenase* (*Gapdh*) and; *18S ribosomal RNA* (*18S*)^46^. qPCR reactions were performed on a CFX96 Real-Time PCR Detection System (Bio-Rad, CA, USA) using EVA Green (Bio-Rad, CA, USA).

**Table 1:** Primer sequences.

Gene	Sequence forward primer (5′–3′)	Sequence reverse primer (5′–3′)
*Hprt*	GCTGGTGAAAAGGACCTCT	CACAGGACTAGAACACCTGC
*Gapdh*	GGGCCCACTTGAAGGGTGGA	TGGACTGTGGTCATGAGCCCTT
*18S RNA*	GTAACCCGTTGAACCCCATT	CCATCCAATCGGTAGTAGCG
*Ifn-γ*	CAACAGCAAGGCGAAAAAGG	GGACCACTCGGATGAGCTCA
*Tnf*	TGCCTATGTCTCAGCCTCTTC	GAGGCCATTTGGGAACTTCT
*Il-1β*	GTGCTGTCGGACCCATATGAG	CAGGAAGACAGGCTTGTGCTC
*Il-6*	CCGGAGAGGAGACTTCACAG	TCCACGATTTCCCAGAGAAC
*Tgf- β*	AGCCCGAAGCGGACTACTAT	AGCCCTGTATTCCGTCTCCT
*Il-10*	AGGACTTTAAGGGTTACTTGGGTT	GCTCCACTGCCTTGCTCTTATT
*Ido*	GGCTTCTTCCTCGTCTCTCTATTG	TGACGCTCTACTGCACTGGATAC
*iNos*	CTCGGAGGTTCACCTCACTGT	GCTGGAAGCCACTGACACTT

### Corticosterone measurement

Sera corticosterone levels were measured 3 days after the last behavioral test. Blood was collected from the tip of the tail within the first 2 min after animals were removed from their home cage. Blood collection occurred between 9 and 10 a.m., corresponding to the beginning of the light period (the basal time-point of the corticosterone production circadian rhythm). Corticosterone concentration was assessed using a radioimmunoassay (RIA) assay kit (Corticosterone Double Antibody RIA kit, MP Biomedicals, NY, USA), following the manufacturer’s guidelines. The detection limit of the assay was 15.4 ng/mL.

### Hippocampal cell proliferation: immunohistochemistry and stereological analysis

To analyze hippocampal cell proliferation, mice anesthetized with a combination of ketamine 75 mg kg^−1^ and medetomidine 1 mg kg^−1^ were transcardially perfused with saline, euthanized by decapitation and their brains removed. Brains were embedded in optimum cutting temperature compound and snap-frozen to assess cell proliferation in the dentate gyrus (DG) using stereological analysis. Serial coronal 20 μm sections were cut on a cryostat, extending over the entire length of the hippocampus. To detect Ki67, a nuclear protein expressed in all phases of the cell cycle except the resting phase G0, a mouse monoclonal anti-Ki67 (Novocastra, UK; 1:100 dilution) was used accordingly with standard procedures. The primary antibody was detected using the Ultravision Quanto Detection System (Lab Vision, CA, USA), and the reaction developed with 3,3′-diamino-benzidine substrate (Sigma Aldrich, MO, USA; DAB: 0.025% and 0.15% H_2_O_2_ in Tris–HCl 0.05 M, pH 7.2). Sections were then counterstained with hematoxylin.

Hippocampal cell proliferation was measured by counting the cells expressing Ki-67 in the subgranular zone (SGZ), considered as the 3-cell-body-wide zone at the border of the DG and normalized by the respective area (results are presented as number of Ki67^+^ cells per mm^2^). The use of the visiopharm integrator system software (Visiopharm, Denmark) allowed the delimitation, at low magnification (40×), of the areas of interest and the identification of the Ki67^+^ cells within the defined areas was performed at higher magnification (400×). Counts were performed by one researcher blind to the experimental conditions.

### Dendritic structure

To analyze the dendritic structure, the mouse brains were collected as described above, immersed in Golgi-Cox solution, and kept in the dark for 14 days at room temperature^47^. The brains were transferred to a 30% sucrose solution and cut on a vibratome. Coronal sections (200 µm thick) were collected in 6% sucrose and blotted dry onto gelatin-coated microscope slides. They were subsequently alkalinized in 18.7% ammonia, developed in Dektol (Kodak, Rochester, NY, USA), fixed in Kodak Rapid Fix, dehydrated, xylene cleared, mounted and coverslipped with Entellan. All incubation steps were performed in a dark room. To minimize bias, each brain was coded to keep the experimenter blind to the experimental conditions. The arrangement of the dendritic material in the granule cells from the DG of the hippocampus was analyzed taking into consideration the following criteria: (1) full Golgi-impregnation along the dendritic tree; (2) complete dendrites without truncated branches; and (3) relative isolation from neighboring impregnated neurons, astrocytes, or blood vessels to avoid interference with the analysis. Slides containing the region of interest were randomly searched and the first 5 to 8 neurons fulfilling the criteria (maximum of 3 neurons per section) were selected.

For each selected neuron, all branches of the dendritic tree were reconstructed, at high magnification (600×), using a motorized microscope (Axioplan 2; Carl Zeiss, Germany) and the Neurolucida software (v. 2019.1.3, MBF Bioscience, Microbrightfield, Inc, VT, USA). A 3D version of a Sholl analysis^48,49^ of the reconstructed neurons was performed using the Neurolucida Explorer software (v. 2019.2.1, MBF Bioscience, Microbrightfield, Inc.; VT, USA); the number of intersections of dendrites with concentric spheres positioned at radial intervals of 10 µm was counted. Additionally, the total length of the dendritic tree was measured. For each group of 5 mice per strain, 30 dentate granule cells were analyzed.

### Data analyses

Normal distribution of the variables was assessed using the Shapiro–Wilk test (p > 0.05). To compare infected with non-infected mice, the independent-sample t-test was performed for variables with normal distribution.

One-way ANOVA was used to compare the bacterial load of the 3 mouse strains. The differences between the groups were analyzed using the Tukey post-hoc test. The Sholl analysis of the reconstructed neurons was performed by ANOVA repeated measures.

The Cohen’s d (*d*) effect size was calculated for all the statistical tests performed considering *d* < 0.50 a small; 0.50 ≤ *d* > 0.8 a medium; and ≥ 0.80 a large effect size^50^.

The results shown are expressed as mean + SEM and correspond to 1 of at least 2 independent experiments with similar results. Sample sizes are shown in the figure legends.

All images were generated using GraphPad Prism software (v.8, GraphPad software Inc. CA, USA). The data were analyzed using GraphPad Prism software (v.8, GraphPad software Inc. CA, USA) or SPSS statistics software (v.27, SPSS Inc. IL, USA). Significance at p < 0.05 is indicated by an asterisk (*).

## Results

### Chronic infection with *Mycobacterium avium* induces a distinct hippocampal cytokine expression profile in the three mouse strains

To evaluate the impact of chronic infection on the neuronal plasticity and behavior of mice we analyzed the animals at 4 wpi with *M. avium*, since it corresponds to the peak of the immune response^17^. We infected mice from 3 mouse strains (Balb/c, C57BL/6 and CD1) also used in other studies that assessed the impact of mycobacteria infection in the brain and behavior. Moreover, these mouse strains are the most widely used in immunological and behavioral studies.

**Figure 1:**
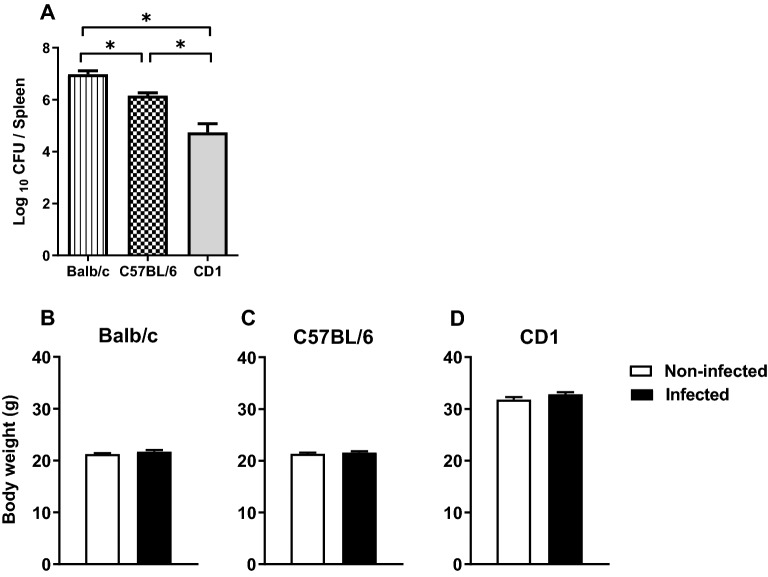
*M. avium* chronic infection does not alter mice body weight even though different susceptibilities to infection are observed. Spleen bacterial load was determined at 4wpi (**A**). Body weight of Balb/c (**B**), C57BL/6 (**C**) and CD1 (**D**) female mice were assessed in non-infected and 4 weeks infected mice. Each bar represents the mean + SEM of 15–24 mice per group, from 1 of 3 independent experiments. *p < 0.05.

The bacterial loads in the spleen at 4 wpi differ among the mouse strains (Fig. 1A; F_(2,57)_ = 530,9; p < 0.0001, *d* = 0.234). Interestingly, Balb/c mice are the most susceptible whereas CD1 mice are the most resistant to bacteria growth. While the bacterial load is very high in the spleen and several other organs^15,18^, very few bacteria are present in the brain. In CD1 mice we detected an average of 0.3 CFU per brain (with 5/7 mice below detection limit), in C57BL/6 mice of 9 CFU per brain (with 5/14 below detection limit) and of 38 CFU per brain in Balb/c mice (with 1/8 below detection limit).

None of the mouse strains revealed weight loss after infection (Fig. 1B–D; Balb/c: t(32) = − 1.667, p = 0.105, *d* = 0.581; C57BL/6 t(43) = − 0.668, p = 0.508, *d* = 0.200; CD1: t(31) = − 1.164, p = 0.253, *d* = 0.402).

**Figure 2:**
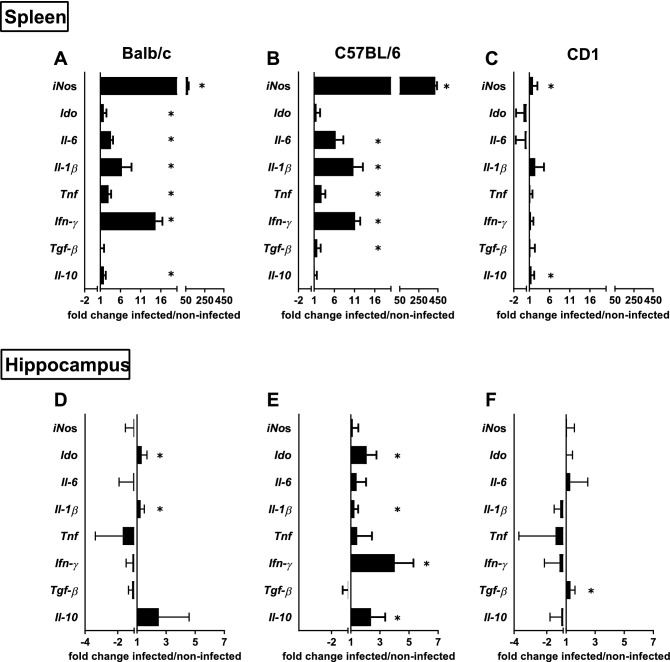
Chronic infection with *M. avium* induces a distinct cytokine/inflammatory molecules profile in the hippocampus and spleen of the different mouse strains. mRNA expression levels for the anti-inflammatory cytokines *Il-10 and Tgf-β,* pro-inflammatory cytokines *Ifn-γ*, *Tnf*, *Il-1β* and *Il-6,* and the inflammatory molecules *Ido* and *iNos* were measured in the spleen (**A**–**C**) and hippocampus (**D**–**F**) of Balb/c (**A** and **D**), C57BL/6 (**B** and **E**) and CD1 (**C** and **F**) mice at 4wpi and non-infected. The mRNA expression levels were normalized using 3 reference genes *Hprt, Gapdh and 18S*. Each bar represents the mean of the fold change of the ratio infected/non-infected + SEM of 6–8 mice per strain from 1 of 2 independent experiments with similar results. *p < 0.05.

The cytokine/inflammatory molecules expression profile in the spleen varies with the mouse strain (Fig. 2A–C). All mice present an increased expression of *iNos*, even though much more exacerbated in C57BL/6 mice [Fig. 2B; t(14) = − 4.942, p < 0.001, *d* = 2.558; Balb/c: Fig. 2A; t(14) = − 6.035, p < 0.001, *d* = 3.123 and CD1: Fig. 2C; t(14) = − 2.271, p = 0.039, *d* = 1.175]. The pro-inflammatory cytokines *Ifn-γ, Tnf, Il-1β and Il-6* are increased in infected Balb/c and C57BL/6 mice, but not in CD1 mice (Fig. 2A,B; *Ifn-γ :* Balb/c: t(13) = − 8.835, p < 0.001, *d* = 4.723; C57BL/6: t(13) = − 7.949, p < 0.001, *d* = 4.249; *Tnf*: Balb/c: t(13) = − 7.671, p < 0.00, *d* = 4.100; C57BL/6: t(14) = − 4.184, p = 0.001, *d* = 2.165*; Il-1β:* Balb/c: t(13) = − 3.524, p = 0.004, *d* = 1.889; C57BL/6: t(14) = − 4.206, p = 0.001, *d* = 2.177 and *Il-6* (Balb/c: t(12) = − 13.191, p < 0.001 , *d* = 7.339; C57BL/6: t(14) = − 3.625, p = 0.003, *d* = 1.876). Infected Balb/c mice also present higher expression levels of *Ido* and *Il-10,* whereas infected C57BL/6 mice present increased *Tgf-β* mRNA expression (Fig. 2A; *Ido:* t(13) = − 4.744, p < 0.001, *d* = 2.536); *Il-10* (t(14) = − 6.119, p < 0.001, *d* = 3.167*; Tgf-β:* Fig. 2B; t(14) = − 2.332, p = 0.035, *d* = 1.207). In the spleen of infected CD1 mice, the only cytokine that present a slight increase when compared with non-infected animals is *Il-10* (Fig. 2C; t(13) = − 2.321, p = 0.037, *d* = 1.241)*.*

The same analysis in the hippocampus revealed that the 3 mouse strains present a distinct cytokine expression profile (Fig. 2D–F). The hippocampus of infected Balb/c and C57BL/6 mice present an increase in *Ido* mRNA expression (Fig. 2D,E; Balb/c: t(14) = − 2.586, p = 0.022, *d* = 1.338; C57BL/6: t(13) = − 3.420, p = 0.005, *d* = 1.828) and *Il-1β* (Balb/c: t(13) = − 2.696, p = 0.018, *d* = 1.441; C57BL/6: t(14) = − 2.640 p = 0.019, *d* = 1.366). Moreover infected C57BL/6 mice also show increased expression of *Ifn-γ* (Fig. 2E; t(14) = − 4.241; p = 0.001, *d* = 2.195) and *Il-10* (Fig. 2E; t(8) = − 2.423; p = 0.042, *d* = 1.625). The infected CD1 mice only present increased expression of *Tgf-β* (Fig. 2F; t(13) = − 2.635, p = 0.021, *d* = 1.408). No differences between infected and non-infected animals are observed for the other cytokines/inflammatory molecules analyzed in the hippocampus (*Il-6, iNos* and *Tnf*).

### *Mycobacterium avium* chronic infection does not impact on hippocampal cell proliferation

**Figure 3:**
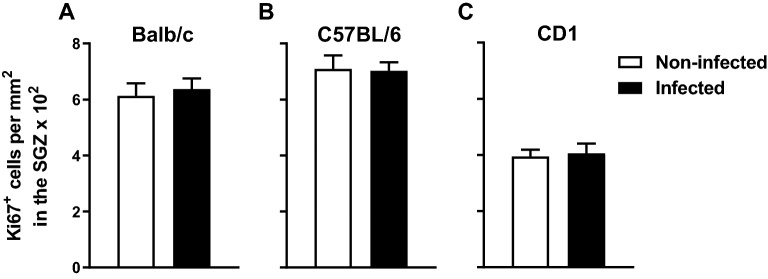
Chronic infection with *M. avium* does not alter the hippocampal cell proliferation. Hippocampal cell proliferation was assessed in *M. avium* infected and non-infected Balb/c (**A**) C57BL/6 (**B**) and CD1 (**C**) mice. The bars represent the mean + SEM of Ki67 + cells per mm^2^ in the SGZ of each animal (6–8 mice per strain from 2 independent experiments).

Since it has been described that inflammation can impact hippocampal neurogenesis^51,52^, we next evaluated the hippocampal cell proliferation in the DG of infected and non-infected animals evaluating the expression of Ki67. *M. avium* infection does not induce alterations in hippocampal cell proliferation in any of the mouse strains analyzed Fig. 3 (Balb/c: t(14) = − 0.470, p = 0.690, *d* = 0.243; C57BL/6 t(10) = 0.113, p = 0.912, *d* = 0.068; CD1: t(13) = − 0.258, p = 0.8, *d* = 0.138).

### *Mycobacterium avium* chronic infection does not affect neuronal plasticity

**Figure 4:**
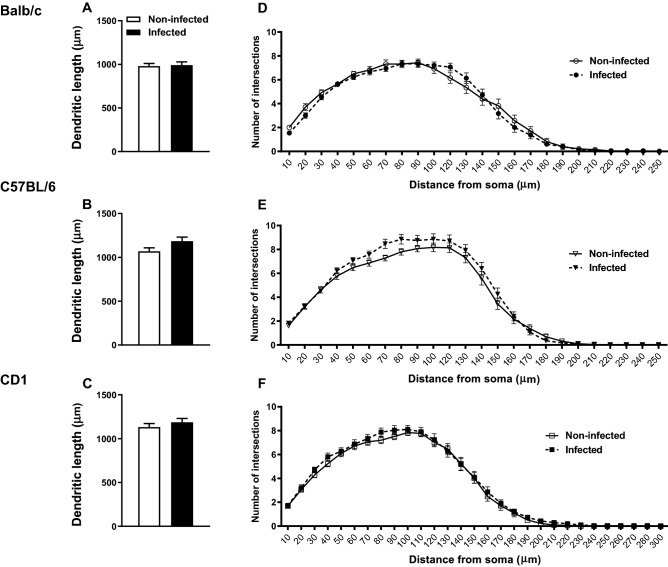
Infection with *M. avium* does not induce morphological alterations in granule neurons of the hippocampus. Dendritic morphology of granule neurons from the DG was analyzed in *M. avium* infected (4 wpi) and non-infected Balb/c, C57BL/6 and CD1 mice. The total length of the dendritic tree (**A**–**C**) and Sholl analysis of the number of intersections of dendrites at specific distances from the soma are displayed (**D**–**F**). Each bar represents the mean + SEM of the average length of 28–31 granule neurons per group, from 5 to 6 mice per group; the lines represent the average number of intersections of dendrite branches with consecutive 10 µm-spaced concentric spheres of the same neurons.

Even though no alterations were observed in hippocampal cell proliferation of infected animals, we assessed whether infection impacts the neuronal morphology of this brain region. The morphological analysis of the DG’s granule neurons revealed that infection with *M. avium* does not induce alterations in the total length of the dendrites from the 3 mouse strains (Fig. 4A–C; Balb/c: t(9) = 1.379, p = 0.201, *d* = 0.872; C57BL/6: t(8) = − 1.215, p = 0.259, *d* = 0.815; CD1: t(8) = − 0.589, p = 0.572, *d* = 0.395). Moreover, the arrangement of the dendritic material of these same neurons, assessed by the number of intersections of dendrites as a function of their distance from the soma, also does not reveal differences between infected and non-infected animals from the 3 mouse strains (Fig. 4D–F; Balb/c: F_(1, 56)_ = 0,2438, p = 0,6234, *d* = 0.132; C57BL/6: F_(1, 55)_ = 2,074, p = 0,1555, *d* = 0.388; CD1: F_(1, 59)_ = 0,8533, p = 0,3594, *d* = 0.241).

### The basal corticosterone levels are unaltered upon *Mycobacterium avium* chronic infection

**Figure 5:**
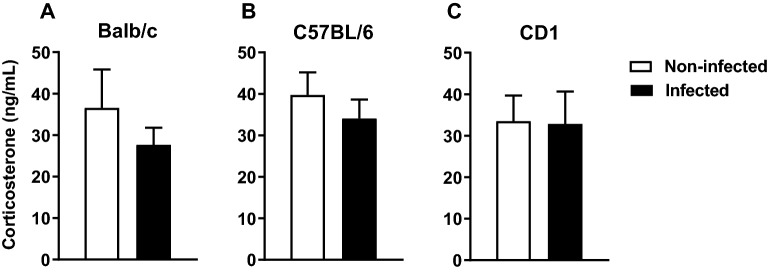
No alterations in basal corticosterone levels were observed in *M. avium* infected compared to non-infected mice. Basal serum corticosterone levels were measured in Balb/c (**A**), C57BL/6 (**B**) and CD1 (**C**) mice non-infected and infected with *M. avium* (4wpi). Each bar represents the mean + SEM from 15 to 16 mice per group from 1 of 2 independent experiments.

Alterations in cytokine levels, namely pro-inflammatory cytokines have been associated with the activation of the hypothalamic–pituitary-adrenal (HPA) axis. Thus, we also analyzed the basal levels of corticosterone. Infection does not induce alterations in the basal levels of corticosterone in the 3 mouse strains (Fig. 5; Balb/c: t(26) = 1.073, p = 0.293, *d* = 0.413; C57BL/6: t(29) = 0.544, p = 0.590,* d* = 0.199; CD1: t(29) = 0.069, p = 0.946,* d* = 0.025).

### *Mycobacterium avium* chronic infection does not induce alterations in locomotor, exploratory, anxious-like or depressive-like behaviors

**Figure 6:**
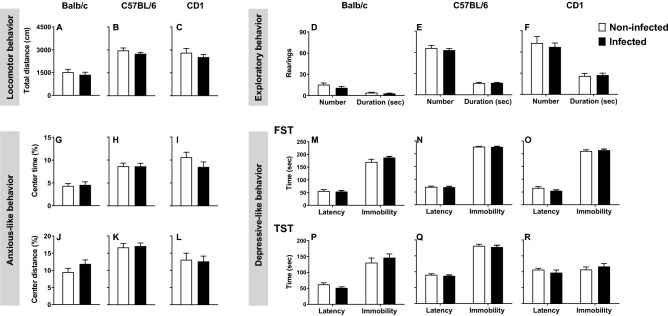
*M. avium* chronic infection does not induce alterations in locomotor, exploratory, anxious-like or depressive-like behaviors. The OF, FST and TST tests were performed with non-infected and infected (4wpi) Balb/c, C57BL/6 and CD1 female mice. In the OF arena, the total distance travelled in centimeters (**A**–**C**), the number and duration of rearings (**D**–**F**), the percentage of time in the center of the arena (**G**–**I**) and center distance (**J**–**L**) were scored**.** In the FST (**M**–**O**) and TST (**P**–**R**) the latency until the first immobility and duration of the immobility periods were assessed. Each bar represents the mean + SEM of 15–23 mice per group, from 1 of 3 independent experiments. *P < 0.05.

To assess the role of a chronic infection in mouse behavior we first analyzed the locomotor and exploratory behavior of the 3 mouse strains in the OF arena. The locomotor and exploratory abilities are not altered by *M. avium* infection in any mouse strain, as measured by the total distance travelled (Fig. 6A–C; Balb/c: t(32) = 0.672, p = 0.506, *d* = 0.234; C57BL/6: t(41) = 1.216, p = 0.231, *d* = 0.375; CD1:t(31) = 0.875, p = 0.388, *d* = 0.312) and the number/duration of the rearings (Fig. 6D–F; number of rearings: Balb/c: t(31) = 1.416, p = 0.167, *d* = 0.501; C57BL/6: t(41) = 0.660, p = 0.513, *d* = 0.204; CD1:t(30) = 0.525, p = 0.604, *d* = 0.192; duration of rearings: Balb/c: t(31) = 1.264, p = 0.216, *d* = 0.447; C57BL/6: t(41) = − 0.184, p = 0.855, *d* = 0.057; CD1:t(30) = − 0.271, p = 0.788, *d* = 0.097). To evaluate anxious-like behavior we measured the time and distance spent in the center of the OF. *M. avium* infection does not alter anxious-like behavior in any of the mouse strains (Fig. 6G–I; center time: Balb/c: t(30) = − 0.231, p = 0.819, *d* = 0.083; C57BL/6: t(41) = 0.025, p = 0.981, *d* = 0.008; CD1:t(30) = 1.387, p = 0.176, *d* = 0.498; Fig. 6J–L; center distance: Balb/c: t(30) = − 1.478, p = 0.150, *d* = 0.531; C57BL/6: t(41) = − 0.260, p = 0.796, *d* = 0.080; CD1:t(30) = 0.202, p = 0.841, *d* = 0.073). To assess the role of chronic infection in depressive-like behavior, mice were submitted to the FST (Fig. 6M–O) and TST (Fig. 6P–R). In both tests no alterations were observed in infected compared with non-infected mice (FST: Fig. 6M–O; latency time: Balb/c: t(30) = 0.868, p = 0.392, *d* = 0.312; C57BL/6: t(39) = 0.214, p = 0.832, *d* = 0.068; CD1:t(30) = 1.702, p = 0.099, *d* = 0.612; immobility time: Balb/c: t(30) = − 1.780, p = 0.085, *d* = 0.640; C57BL/6: t(39) = − 0.168, p = 0.867, *d* = 0.053; CD1:t(30) = − 0.512, p = 0.612, *d* = 0.184); (TST: Fig. 6P–R; latency time: Balb/c: t(30) = 1.801, p = 0.082, *d* = 0.647; C57BL/6: t(39) = 0.706, p = 0.484, *d* = 0.223; CD1:t(29) = 0.897, p = 0.377, *d* = 0.328; immobility time: Balb/c: t(30) = − 0.843, p = 0.406, *d* = 0.303; C57BL/6: t(39) = 0.507, p = 0.615, *d* = 0.160; CD1:t(29) = − 0.749, p = 0.460, *d* = 0.273).

## Discussion

Over the last decades, several studies demonstrated a bidirectional interaction between the immune and central nervous systems. An imbalance in the immune system is often associated with alterations in behavioral phenotypes. Taking into account the high prevalence of chronic infections, namely those caused by bacteria from the *Mycobacterium* genus^10^, we investigated whether exposure to a chronic infection with *M. avium 2447*, during which the cytokine expression profile is continuously altered, triggers behavioral alterations in various mouse strains. Upon infection with *M. avium*, a mouse strain specific cytokine expression profile is observed both in the spleen and in the hippocampus. However, this imbalanced cytokine *milieu* does not impact corticosterone production, hippocampal cell proliferation or the DG’s granule neurons morphology. The chronic infection with *M. avium* does not induce alterations in locomotor, exploratory, anxious, or depressive-like behaviors. Similar behavioral results were observed after 12 weeks of intravenous infection in the 3 mouse strains (with 10^6^ CFU; supplementary Fig. S1), 4 weeks of *M. avium* intraperitoneal infection (with 10^7^ CFU) in C57BL/6 mice and after 4 weeks of intravenous infection (with 10^7^ CFU) in Wistar rats (data no shown). We also analyzed the effects of intravenous *M. avium* 2447 infection in male mice at 4 and 12 weeks using the two mouse strains, Balb/c and C57BL/6. No differences in susceptibility to infection compared to females were observed, nor were there changes in behavioral phenotype when comparing infected and non-infected animals (data not shown).

We can conclude that even though infection with *M. avium* induces an imbalance in the immune system, it is not associated with alterations in the behavior of the animals nor in neuronal plasticity of the hippocampus. The results also showed several interstrain differences that are in accordance with a previous study^53^. Of notice Balb/c female mice showed a more pronounced anxious-like phenotype whereas C57BL/6 mice present a more depressive-like trait^53^. Furthermore, CD1 mice present the lower number of proliferating cells in the hippocampus, while hippocampal dentate granular neurons of Balb/c mice show smaller dendritic lengths and fewer ramifications^53^.

Infection of mice with *M. avium* 2447 induces an increase in bacterial load until 4 wpi, the peak of the immune response^17^, after which the bacterial load reaches a plateau for several months in organs such as the spleen and the liver^15,18^. Thus, although the host can control bacterial growth, it cannot reduce or eliminate the bacteria within these organs^15,18^. Since infection by mycobacteria results in a prolonged alteration of the cytokine expression profile, namely sustained production of pro-inflammatory cytokines, we considered it to represent a good model to address how chronic infection interferes with mood behavior. The distinct susceptibility of different mouse strains to *M. avium* infection was previously described^18^. Here we also observed that it is accompanied by a different profile of cytokine expression both in spleen and hippocampus. These cytokine alterations, in the periphery and brain, are not associated with alterations in hippocampal plasticity (cell proliferation or morphology in granule neurons of the hippocampus) as usually described^51^. Other authors have also shown that despite the peripheral inflammation observed in a human TNF transgenic mouse model no alterations in hippocampal cytokine *milieu* and cellular plasticity were observed^54^. One can hypothesize that despite of the alteration in the spleen cytokine expression profile, the increase in the cytokines observed in the hippocampus is not sufficient to induce hippocampal plasticity and/or behavioral changes. To our knowledge, these parameters of hippocampal plasticity were not previously investigated in other mycobacterial infections.

The absence of behavioral alterations in the various strains of infected mice, all with an imbalance in the cytokine *milieu* in the spleen and hippocampus, clearly indicates that increased production of pro-inflammatory molecules is insufficient to trigger changes in behavior. This evidence supports the notion that other factors contribute to triggering mood disorders. Previous studies performed in mice infected with BCG showed that infection-induced depression was associated with increased production of TNF and IFN-γ, and with IDO activation^25,26,28,31^. Activation of IDO enzyme has been proposed as one of the events responsible for the transition from a sickness state into a depressive disorder^25,26,28,31^. In the present work, we also observed increased expression levels of *Ido* in the hippocampus of C57BL/6 and Balb/c mice. However, no behavioral alterations were detected which led us to hypothesize that other pathways could be involved. One consistent observation in the BCG infection model is an initial sickness behavior, accompanied by alterations in locomotion and body weight loss that were recovered after a few days^25–31^. However, mice can be infected with *M. avium* 2447 for several months without clinical signs of disease or weight loss. Accordingly, it was also observed that upon *M. avium* infection, besides the absence of alterations in body weight, no variations in body temperature were present^14^.

Supporting the idea that other mechanisms orchestrate with the imbalance of the immune system to induce mood disorders are also the results showing a mouse-to-mouse variation within BCG-inoculated animals^27,29^. Up to 30% of BCG-inoculated mice, despite the evidence of an activated immune system, did not exhibit increased depressive-like behavior and were categorized as ‘‘resilient’’ to BCG-induced behavioral changes^27^. Curiously, the “resilient” group does not present alterations in corticosterone levels whereas the mice “susceptible” to BCG-induced behavioral changes showed elevated plasma corticosterone levels when compared with control animals^27^. The fact that cytokines influence the HPA axis and that increased levels of corticosterone are associated with depression, guides us to hypothesize that the imbalance in the cytokine expression profile should be accompanied by activation in the HPA axis to induce behavioral alterations. In the present study, even though an increased inflammatory profile upon *M. avium* 2447 infection is present, no alterations in corticosterone levels were observed.

The relation between infection and behavioral alterations is far from being understood. It seems to be a complex interplay that could be influenced by several factors such as host, pathogen or environment, and mediated by various mechanisms^55,56^. In the opposite direction of the studies with BCG inoculation, studies with *M. vaccae* showed that this mycobacteria induces decreased depressive-like behavior and stress resilience^32–37^. The mechanisms underlying behavior alterations upon infection might be highly variable. Antidepressant-like behavioral alterations observed after administration of *M. vaccae* antigens are due to activation of a serotonergic subpopulation of neurons within the interfascicular part of the dorsal raphe nucleus^32^ and on the dorsal raphe nucleus, ventrolateral part/ventrolateral periaqueductal gray^57^ and can also be associated with a less inflammatory *milieu* present in the hippocampus^35^.

## Conclusion

In summary, with this study we conclude that peripheral chronic infection with *M. avium* 2447 leads to an altered inflammatory *milieu *in the hippocampus, that does not trigger neuroplasticity nor behavioral alterations in 3 mouse strains. These results highlight that other pathway(s) must synergize with the imbalance of the immune system to trigger mood disorders. HPA axis activation and alterations in neurotransmitter homeostasis, are strong candidates that should be addressed in the future.

## Supplementary Information

Supplementary Figure S1.

## Data Availability

The data supporting the results of this study are available from the corresponding author (S. Roque) upon reasonable request by any qualified investigator for the purpose of replicating the procedures and results.

## Abbreviations

18S: 18S ribosomal RNA
BCG: *Mycobacterium **bovis* Bacillus Calmette-Guérin
CFU: Colony-forming units
DG: Dentate gyrus
FST: Forced swim test
GAPDH: Glyceraldehyde 3-phosphate dehydrogenase
HPRT: Hypoxanthine guanine phosphoribosyl transferase
IFN-γ: Interferon-γ
iNOS: inducible nitric oxide synthase
IDO: Indoleamine 2,3-dioxygenase
IL: Interleukin
OFT: Open field test
SGZ: Subgranular zone
TNF: Tumor necrosis factor
TST: Tail suspension test
wpi: Weeks post infection
