# Supplementary material for: Chronic Mycobacterium avium infection differentially affects the cytokine expression profile of three mouse strains, but has no effect on behavior
Source: Sci Rep. 2023 Apr 17;13:6199. doi: 10.1038/s41598-023-33121-2 (PMC10110542; doi:10.1038/s41598-023-33121-2)
Supplement: Supplementary file 1 — Supplementary Figure S1. [file 41598_2023_33121_MOESM1_ESM.pdf]

Supplementary Information

Supplementary Figure S1

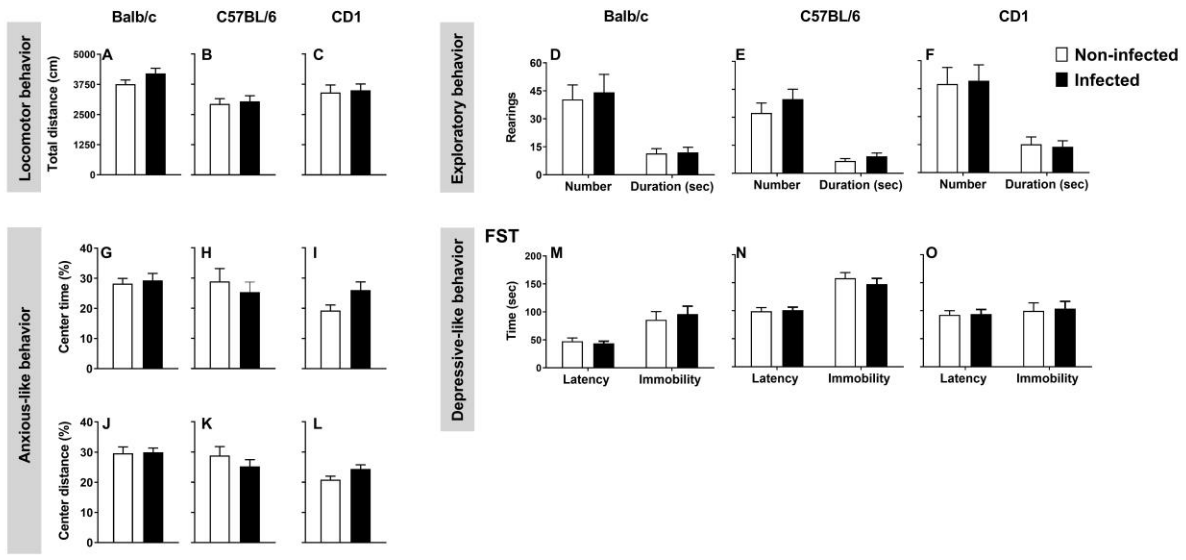

Suplemmentary Fig. S1 - *M. avium* chronic infection does not induce alterations in locomotor, exploratory, anxious-like or depressive-like behaviors at 12 wpi. The OF, and FST tests were performed with non-infected and infected (12 wpi) Balb/c, C57BL/6 and CD1 female mice. In the OF arena, the total distance travelled in centimeters (A, B, and C), the number and duration of rearings (D, E and F), the percentage of time in the center of the arena (G, H, and I) and center distance (J, K and L) were scored**.** In the FST (M, N and O) the latency until the first immobility and duration of the immobility periods were assessed. Each bar represents the mean + SEM of 11-15 mice per group, from 1 of 2 independent experiments.
